# Pharmacokinetic profile of oral and subcutaneous administration of paracetamol in the koala (*Phascolarctos cinereus*) and prediction of its analgesic efficacy

**DOI:** 10.1371/journal.pone.0300703

**Published:** 2024-04-17

**Authors:** Merran Govendir, Larry Vogelnest, Amanda J. Shapiro, Caroline Marschner, Benjamin Kimble

**Affiliations:** 1 Sydney School of Veterinary Science, The University of Sydney, Sydney, Australia; 2 Taronga Conservation Society Australia, Mosman, NSW, Sydney, Australia; Dr. Anjali Chatterji Regional Research Institute for Homeopathy, INDIA

## Abstract

The pharmacokinetic profile of paracetamol in koalas is described when administered orally at 15 mg/kg; followed by the same dose, administered every 12 hours (hrs), repeated five times. After the initial oral administration, the median (range) maximal plasma concentration (C_max_), the time C_max_ was reached (T_max_) and elimination half-life (t_1/2_) were 16.93 μg/mL (13.66 to 20.25 μg/mL); 4 hrs (4 to 8 hrs) and 5.54 hrs (4.66 to 7.67 hrs), respectively. When paracetamol was administered orally at 15 mg/mL every 12 hrs, the trough total plasma concentration range remained comparable to the therapeutic range in humans i.e. 4 to 20 μg/mL that is known to provide some analgesia. However, there is a smaller proportion of free drug (i.e. not bound to plasma proteins; and the active form) available in koala plasma (approximately 40% unbound) compared to human plasma (approximately 80% unbound). Consequently, even when there are similar total drug plasma concentrations in both koala and human plasma, the therapeutic efficacy may be reduced in koalas compared to humans. The initial oral dose and subsequent twice daily doses resulted in no obvious adverse effects in any koala. Haematology, plasma electrolyte and biochemical analyte values remained within their reference ranges eight hrs after the last dose but there was a significant change in alanine transaminase (ALT) levels (an increase), and in total protein (a decrease) (both p = 0.03). A dose of 15 mg/kg was also administered as a subcutaneous injection, diluted 50:50 with saline, to two koalas. As the oral formulation and the subcutaneous administration resulted in comparable absorption, the study focused on the oral profile. Based on these results there is an argument to recommend a slight increase in the oral paracetamol dose for the koala, however further investigation is required to confirm whether repeated administration of a slightly higher dose may be associated with more severe or additional significant changes in haematology, electrolytes or biochemical analytes. However, a preferable recommendation would be to administer this dosage of paracetamol in combination with another analgesic such as tramadol, as a subcutaneous injection, to improve efficacy.

## Introduction

The koala is a revered, iconic, Australian marsupial, but many free-ranging koalas are injured by car strikes, bushfires or animal attacks and are susceptible to infectious diseases such as chlamydiosis, all potentially requiring analgesia to improve patient recuperation and survival. There are a few published pharmacokinetic (PK) profiles for analgesics for the koala such as subcutaneous (s.c.) injection of tramadol [1], and fentanyl as an intravenous bolus injection or 25 μg/hour transdermal patches [2]. Additionally, the non-steroidal anti-inflammatory drug (NSAID) meloxicam has poor oral absorption in the koala and when administered intravenously, has a short elimination half-life of only 1.19 hrs (range 0.71 to 1.62 hrs) [3, 4], compared with 24 hrs in dogs [5] and approximately 13 hrs in humans [6]. Therefore, in contrast to many other species, meloxicam would require frequent dosing which is problematic from the management side and stressful for a wildlife species. All the afore-mentioned medicines are only available via a veterinary prescription for the individual koala. Therefore, there is a need to identify other analgesics that are not only efficacious, available ‘over-the counter’ (i.e. do not require a prescription), but also have a longer duration of analgesia to minimise animal handling.

Paracetamol, also known as acetaminophen in North America and some other countries, is a centrally acting analgesic used in many species and the most popular analgesic world-wide to treat pyrexia, and control mild to severe pain in humans [7]. Its mechanism of action is not completely understood but is reported to act as a cyclo-oxygenase (COX) 2 antagonist in neural tissue, consequently inhibiting the biotransformation of arachidonic acid to prostaglandins [7]. Paracetamol also activates the serotonergic descending inhibitory pathway, the endocannabinoid system and the transient receptor potential vanilloid-1 channel, both latter actions are involved in the modulation of pain signaling [8].

Paracetamol administration, at low doses is potentially fatal to cats. Due to the lack of phase II UDP-glucuronosyltransferase (UGT) enzymes in cats an oral dose from 60 mg/kg body weight (BW) will result in the accumulation of the phase I metabolite N-acetyl-p-benzoquinone-imine (NAPQI) [9]. Accumulation of NAPQI damages red-blood cell membranes, reduces haemaglobin to methaemaglobin and results in hepatocellular damage [10]. Doses greater than 100 mg/kg BW can also induce hepatocellular damage in dogs [11].

Paracetamol +/- codeine has been administrated orally to koalas for decades [12], however there is no information published on paracetamol’s PK profile, it’s efficacy or potential buildup of NAPQI or other metabolites in this species. The aim of this study was to describe the PK profile of paracetamol when administered orally and injected as a single s.c. injection; to predict the likely efficacy, to document the in-vitro metabolism and to identify NAPQI and other metabolites if present. An additional aim was to ascertain if repeated oral dosing at 15 mg/kg every 12 hrs on five occasions resulted in any significant changes in haematology, plasma electrolytes or biochemical analytes.

## Materials and methods

### Animals

Eight, adult, clinically normal koalas (4 males, 4 females) with median (range) body weights and ages of 7.9 kg, (7.2 to 10.3 kg) and 4.9 years (2.8 to 7.5 years), respectively, were recruited from the Taronga Zoo population (Mosman, NSW, Australia). These koalas were clinically normal based on regular physical examinations, and haematology and biochemical analyte values. During the study, koalas were housed individually and supplied with various *Eucalyptus* spp. foliage and water *ad-libitum* as per normal husbandry. This study was approved by the Taronga Conservation Society of Australia, Animal Ethics Committee protocol 3d/06/21.

### Drug administration and blood collection

#### Placement of intravenous catheter for blood collection

The koalas were anaesthetised with alfaxalone (Alfaxan, Jurox, Pty Ltd, Rutherford, NSW, Australia) at 3 mg/kg administered intramuscularly (i.m.) and maintained under anaesthesia on isoflurane in 100% oxygen via a face mask for placement of a 20 gauge, 1 ¼ inch intravenous catheter into the cephalic vein. A short T-connector extension set (Codan, Santa Ana, CA, USA) and cap was attached to the catheter and flushed with heparinised saline. The extension tube, cap and catheter were secured with tape and bandage, for serial blood collection [13]. Blood (5 mL) was collected at the time of anaesthesia in a lithium heparin tube to establish baseline haematology and biochemistry (designated t = 0 hr).

#### Dosing of animals

Paracetamol as an oral suspension (Panadol Children 1 Month– 1 Year 100 mg/mL Cheery Vanilla flavour, GlaxoSmithKline Australia, Ermington, NSW) was administered to two koalas (one of each sex) at a dose of 15 mg/kg. The oral dose was selected because it is the current suggested dose for koalas [14]. Additionally, an injectable paracetamol formulation for intravenous infusions to people (Symbion Paracetamol 1000mg/mL solution, Greystanes, NSW) was injected s.c. at 15 mg/kg, diluted 50:50 with isotonic saline, to another two koalas (one of each sex). As many drugs such as enrofloxacin [15], and meloxicam [3] have superior s.c. absorption compared to the oral route, the s.c. route was initially selected for this study. However, as the resulting paracetamol area under the curve concentrations were similar between the oral and s.c. administration, it was decided to focus on the oral PK profile and therefore the remaining koalas were medicated with the initial oral dose of 15 mg/kg, and after 24 hrs, these koalas were administered the same dose every 12 hrs on five occasions.

#### Blood collection

To determine paracetamol, and the resultant sulphate and glucuronide metabolite plasma concentrations, serial blood samples (up to 2 mL) were collected into lithium heparin tubes at t = 0.25, 0.5, 1, 2, 4, 8 and 12 hrs after drug administration. The cap, extension tube and catheter were flushed with heparinised saline before and after each collection. The first 0.5 mL of blood was discarded to avoid dilution of samples with heparinised saline. A blood sample was collected at 24 hrs just before the next dose, then at 48 and 72 hrs (all “trough” levels i.e. taken prior to the next dose) with the final sample collected at 78 hrs. Samples were centrifuged within 1 hr of collection; the plasma was immediately stored at –80°C and protected from light until drug quantification that occurred within 1 month of collection.

At T = 0 and T = 78 hrs blood was collected in lithium heparin tubes from the six koalas to detect if there were any changes in haematology, biochemical analytes and electrolytes.

### Drug analysis method and sample processing

#### Chemicals

Paracetamol, paracetamol β-D-glucuronide, paracetamol sulphate potassium salt (PS), N-acetyl-benzoquinoneimine (NAPQI), trimethoprim as the internal standard (IS), trifluoroacetic acid (TFA) and all chemicals used for the phase I and phase II *in vitro* assays were purchased from Sigma-Aldrich Pty Ltd (Macquarie Park, NSW). High pressure liquid chromatography (HPLC) grade methanol (MeOH), ammonium acetate, and triethylamine were purchased from Thermo Fisher Scientific (Macquarie Park, NSW, Australia).

#### Source of microsomes

The koala and common brush-tailed possum microsomes were obtained opportunistically from wildlife hospitals when an animal died or euthanased, due to acute, severe trauma such as when struck by vehicles or attacked by feral animals. The method for extraction of the microsomes from the liver of koalas and common brush-tailed possums (*Trichosurus vulpecula*) are described elsewhere [4]. The canine male microsomes (M00201) and feline microsomes (S00846) were obtained from Bioreclamation IVT Frankfurt Germany and BioIVT Baltimore, Maryland USA, respectively.

#### Drug analysis

Quantification of paracetamol, metabolites paracetamol-glucuronide (PG) and paracetamol-sulphate (PS) in plasma by HPLC with UV detection was modified from a previously published method [16]. Briefly, a Shimadzu Nexera XR LC system (Rydalmere, NSW, Australia) was used; A Supelco, Discovery© C 18, (25cm x 4.6mm, 5 um) column as the stationary phase, and maintained at 40°C, was used. The retention times of paracetamol, PG, PS, and IS were 8.17-, 6.08-, 7.55-, and 10.20 min, respectively, which were monitored via a UV-wavelength at 245 nm.

#### Standard curves

Standard curve concentrations, ranging from 0.625 to 20 μg/mL for paracetamol and PG, and 0.625 to 10 μg/mL for PS and quality control samples at a concentration of 0.625, 1.25, 5, 10, and 20 μg/mL for paracetamol, PG and PS were prepared in blank pooled koala plasma collected from a minimum of three koalas. Using a ratio against the IS, standard curves (regression lines) were prepared individually for paracetamol, PG and PS (all with *r*^2^ > 0.99) with / or without using a weighting factor (1/*x*^*2*^). For convenience of the sample preparation, this study set the lower limit of quantification (LLOQ) as 0.625 μg/mL for paracetamol, PG, and PS. This LLOQ was well above the theoretical LLOQ calculated from intercept (SD) and slope (average) of regression lines, and met the criteria for setting the LLOQ (e.g., precision and accuracy were <15% and within 20% of nominal concentration) [17]. Accordingly, the accuracy and precision of the QCs (S1 Table) were within accepted criteria [17] and the absolute recoveries (average ± SD, n = 12) of paracetamol, PG, PS, and IS were 78.86 ± 7.54, 94.72 ± 9.90, 81.92 ± 7.54, and 91.83 ± 5.02, respectively.

#### Extraction

For the mobile phase, 20 mM ammonium acetate (consisted of 0.1% triethylamine) pH 4.5 was prepared as both 4.8% MeOH (Mobile phase A) and 80% MeOH (Mobile phase B). Accordingly, the following gradient was applied for separation of analytes; 0–1 min: maintained at 0% mobile phase B, 1–9 min: increased to 45% mobile phase B, 9–11 min: maintained at 45% mobile phase B, 11–14 min: equilibrium 0% mobile phase B. For drug extraction and sample preparation: 100 μL of 20% TFA (which contained the IS at a concentration of 1 mg/mL in MeOH) was added to 100 μL of the plasma samples collected from koalas administered paracetamol, the plasma standards prepared for the standard curves and the QC samples. All samples were vortexed and centrifuged at 14000 g for 10 minutes and 10 μL of the supernatant was injected into the HPLC system.

#### Pharmacokinetic analysis

Paracetamol concentrations only, underwent non-compartmental analysis. The peak concentration (C_max_) and the time this was reached (T_max_) was obtained directly from the measured concentrations. The elimination half-life (t_1/2_) was determined by ln2/k_e_ where k_e_ is the elimination rate constant (the inverse slope of the elimination or terminal part of the semi-log curve). The absorption constant k_a_ was determined by the method of residuals [18]. The area under the concentration-time curve (AUC_0-24_) was calculated to the measurable concentration at 24 hrs (time ‘t’ in equations) using the log-linear trapezoidal method using Monolix https://monolix.lixoft.com. The AUC and AUMC from the last observed concentration to infinity were determined by:

$${\mathrm{AUC}}_{t‐\infty }=C_{\mathrm{last}}/k_{e}$$

$$AUMC_{t‐\infty }=(C_{\mathrm{last}}\times t_{\mathrm{last}}/k_{e})+(C_{\mathrm{last}}/{k_{e}}^{2})$$

The mean residence time (MRT), clearance (Cl/F), apparent volume of distribution (V/F) were determined by the following equations:

$$\mathrm{MRT}={\mathrm{AUMC}}_{0‐\infty }/{\mathrm{AUC}}_{0‐\infty }$$

$$\mathrm{Cl}/F=\mathrm{Dose}/{\mathrm{AUC}}_{0‐\infty }$$

$$V/F=Cl/k_{e}$$

F = bioavailability, which could not be calculated.

PK Solver [19] was used to undertake the non-compartmental analysis.

To ascertain if there was any paracetamol increase in peak concentration 48 hrs versus 72 hrs after administration the accumulation factor was used:

$$\left(1-e^{-nk\tau })/(1-e^{-k\tau }\right)$$

Where n = the number of doses after the baseline dose

k = k_e_ = elimination constant

τ = number of hrs between dosing

#### Paracetamol binding to plasma proteins

The percentage of paracetamol bound to koala plasma proteins was determined using the ultrafiltration method [20] based on a modified protocol [3]. Paracetamol at the following concentrations of 15 μg/mL (4 replicates), 30 μg/mL (2 replicates) and 150 μg/mL (2 replicates) were added to 1 mL of initially frozen, thawed pooled blank koala plasma, adjusted to pH 7.4, and incubated in a water bath at 37°C for 30 min. Then 200 μL of plasma was removed for determination of the total concentrations (Drug _total_) and the remaining plasma was transferred to the reservoir of the Centrifree ultrafiltration device (Merk Millipore, Macquarie Park, Australia) with a membrane molecular weight cut‐off of 30 kDa. The ultrafiltration device was centrifuged at 1,500 x *g* for 10 min at 37°C. After centrifugation, the filtrate was used to determine the free concentrations (Drug _free_). Both the unbound and total fractions were quantified by HPLC as described above. The percentage of substrate binding to plasma proteins was estimated as 100 - [(Drug _free_/Drug _total_) × 100]. The same concentrations of paracetamol were added to phosphate buffer saline and underwent ultrafiltration to determine the non-specific binding to the filtration membrane.

### In vitro microsome studies to identify the presence of NAPQI and the rate of paracetamol-glucuronide formation

As paracetamol can undergo both phase I metabolism and phase II metabolism, *in vitro* assays using hepatic enzymes (microsomes) were undertaken to ascertain to compare the paracetamol depletion rate and appearance of metabolites using koala microsomes and those from other species.

### Phase I in vitro study

This method is reported elsewhere [4] and involves paracetamol (1 μM or 50 μM) being pre-incubated in 3 mL of 0.1 M phosphate buffer (pH 7.4) containing a nicotinamide adenine dinucleotide phosphate (NADPH) regenerating system (1 mM NADP, 0.8 U glucose 6 phosphate dehydrogenase and 3 mM glucose 6 phosphate) and 3 mM MgCl_2_, in an open air shaking water bath at 37°C for 5 min. The enzymatic reaction was then initiated by adding a predetermined concentration of common brush-tailed possum microsomal protein (0.25 mg/mL) during the incubation and 1 mL aliquots were removed at *t* = 0, 20, and 30 min and then analysed by the HPLC method described above.

#### Phase II in vitro glucuronidation assay

The substrate depletion method was modified from that of Slovak et al., 2017 [21], whereby paracetamol (2.5 μM) was preincubated in 1.0 mL of 0.1 M phosphate buffer (pH 7.4) containing 2.5 μg/μL alamethicin with 0.5 mg/mL of pooled microsomes from the following species: koala (incorporating microsomes from two females), the common brush-tailed possum (three individuals), male dogs (seven individuals) and male cats (three individuals) in an open air shaking water bath at 37°C for 3 min. After pre-incubation, glucuronidation was initiated by adding a 100 μL of the cofactor solution that contained 100 mM potassium phosphate buffer, 50 mM uridine diphosphate glucuronic acid (UDPGA) and 50 mM MgCl_2_. During incubation, 200 μL aliquots were removed at t = 0, 30, and 60 min. 50 μL of 10% of TFA in methanol was added to each aliquot, vortexed, centrifuged (14,000 x g for 10min), and 20 μL of clear solution was injected for quantification.

For identification of paracetamol glucuronide metabolite 5, 10, 50, 100, and 1000 μM of paracetamol was incubated with 0.5 mg/mL of the microsomes mentioned in the previous paragraph in 1mL of 100 mM potassium phosphate buffer (pH 7.4), containing 25 μg of alamethicin, in an open-air shaking water bath at 37°C for 30, 60, or 90 min and 200 μL aliquots were removed at these time-points. Liquid-liquid extraction, using ethyl acetate, was used for sample preparation.

The same HPLC conditions were used to detect PG as described above, however an isocratic mobile phase was used and consisted of a mixture of 0.1M sodium acetate buffer (pH 3.6) and MeOH (120:10, v/v) at a flow rate of 1.0 mL/min. The injection volume was 20 μL. The UV wavelength was 245 nm.

The *in vitro t*_1/2_ and *in vitro* Cl_int_ were calculated by the substrate depletion method utilizing an *in vitro t*_1/2_ approach [22]. For the rate of depletion, the paracetamol at t = 0 was considered 100% of substrate, the change in paracetamol concentration over time were converted to a percentage of the substrate remaining. Further, this was plotted as natural log of remaining paracetamol vs. incubation time, and the slope of the regression line, represented as a constant rate (-k) was used for to calculate t_1/2_ (min) and *in vitro* Clint (μL/min/mg protein) according to the following equations:

$$In\;vitro\;t_{1/2}=‐0.693/k$$

*In vitro* Cl_int_ = (0.693 / in vitro t_1/2_) x (μL incubation volume / mg protein)

k = slope of depletion

### Statistical analyses

Each electrolyte and biochemical analyte value were compared between the value at T = 0 and 78 hrs for six koalas. Likewise for the haematology, haematocrit %, haemoglobin concentration, white blood cell counts were also compared at these time points. Many of these values did not have equal variances and therefore underwent statistical comparison by the non-parametric Wilcoxon matched pairs signed rank test with level of significance set at p < 0.05.

## Results

The accuracy and precision of the assay based on the QC samples’ plasma concentrations of paracetamol, P-G and P-S in koala plasma on three occasions is provided in S1 Table. S2 Table provides the sexes, weights, and ages of all koalas.

There were no statistical differences in any of the haematological values; the median (range) haematocrit value at T = 0 hr was 31% (range 30 to 36%) and at t = 78 hrs was 30.5% (25 to 34%).

**Fig 1:**
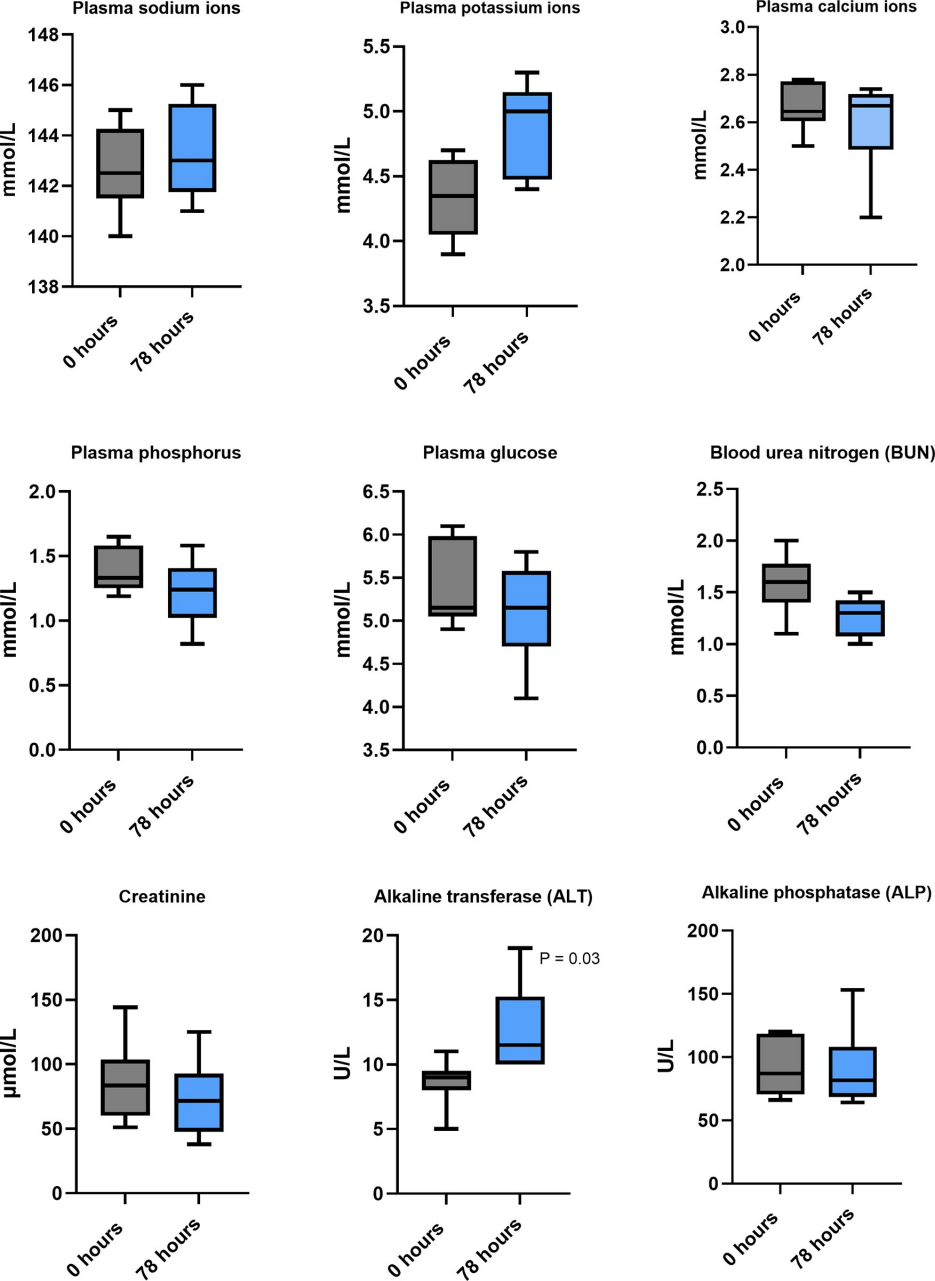
Range of plasma electrolyte and biochemical analyte values at t = 0 and 78 hours when paracetamol was dosed orally at 15 mg/kg twice daily in six koalas. Wilcoxon matched pairs signed rank tests demonstrated a significant difference in ALT only (p = 0.03).

**Fig 2:**
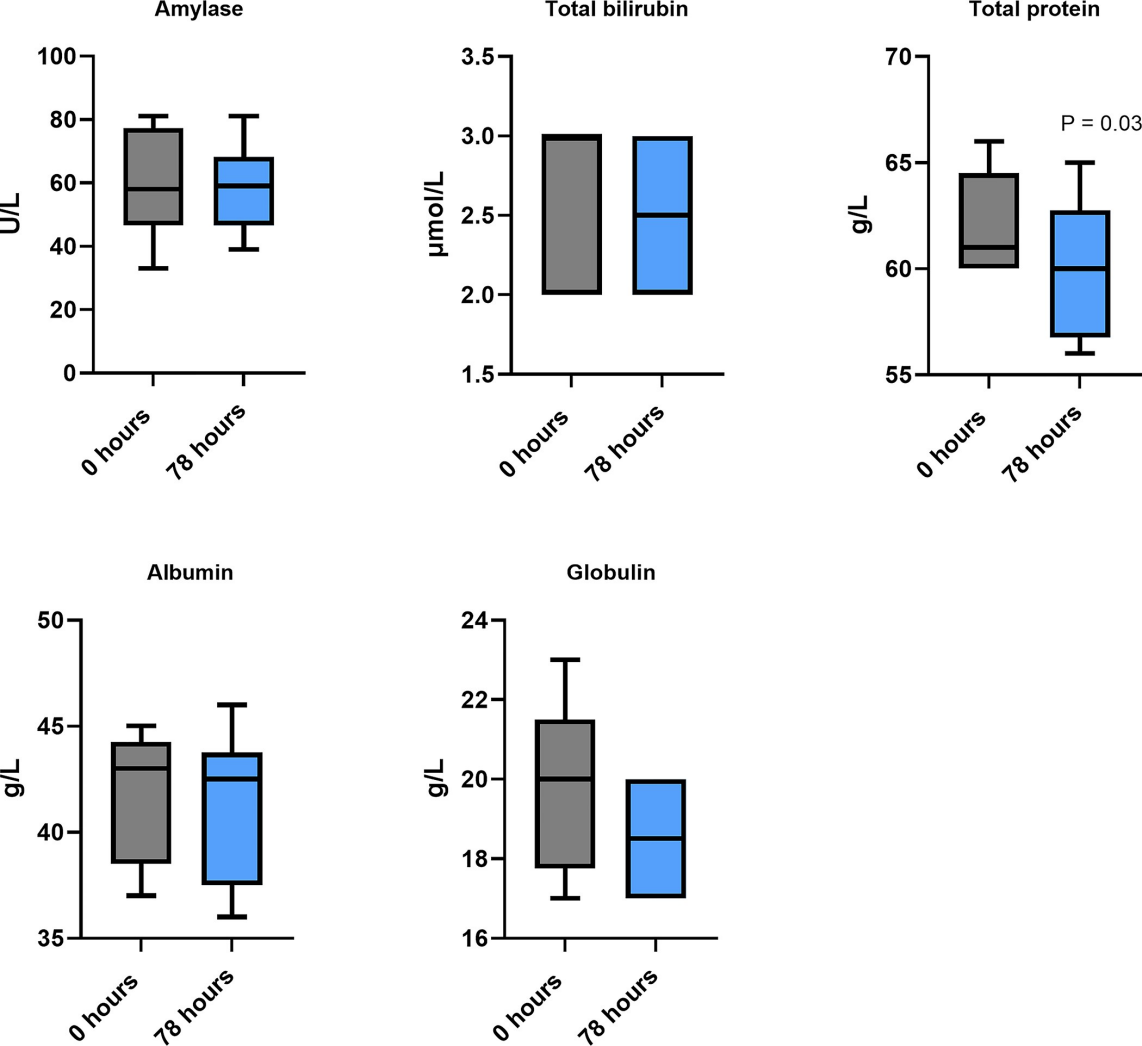
Range of plasma electrolyte and biochemical analyte values at t = 0 and 78 hours when paracetamol was dosed orally at 15 mg/kg twice daily in six koalas. Wilcoxon matched pairs signed rank tests demonstrated a significant difference in total protein only (p = 0.03).

The changes in electrolytes and biochemical analytes when paracetamol was dosed orally at 15mg/kg twice daily at T = 0 and 78 hrs are provided in Figs 1 and 2.

**Fig 3:**
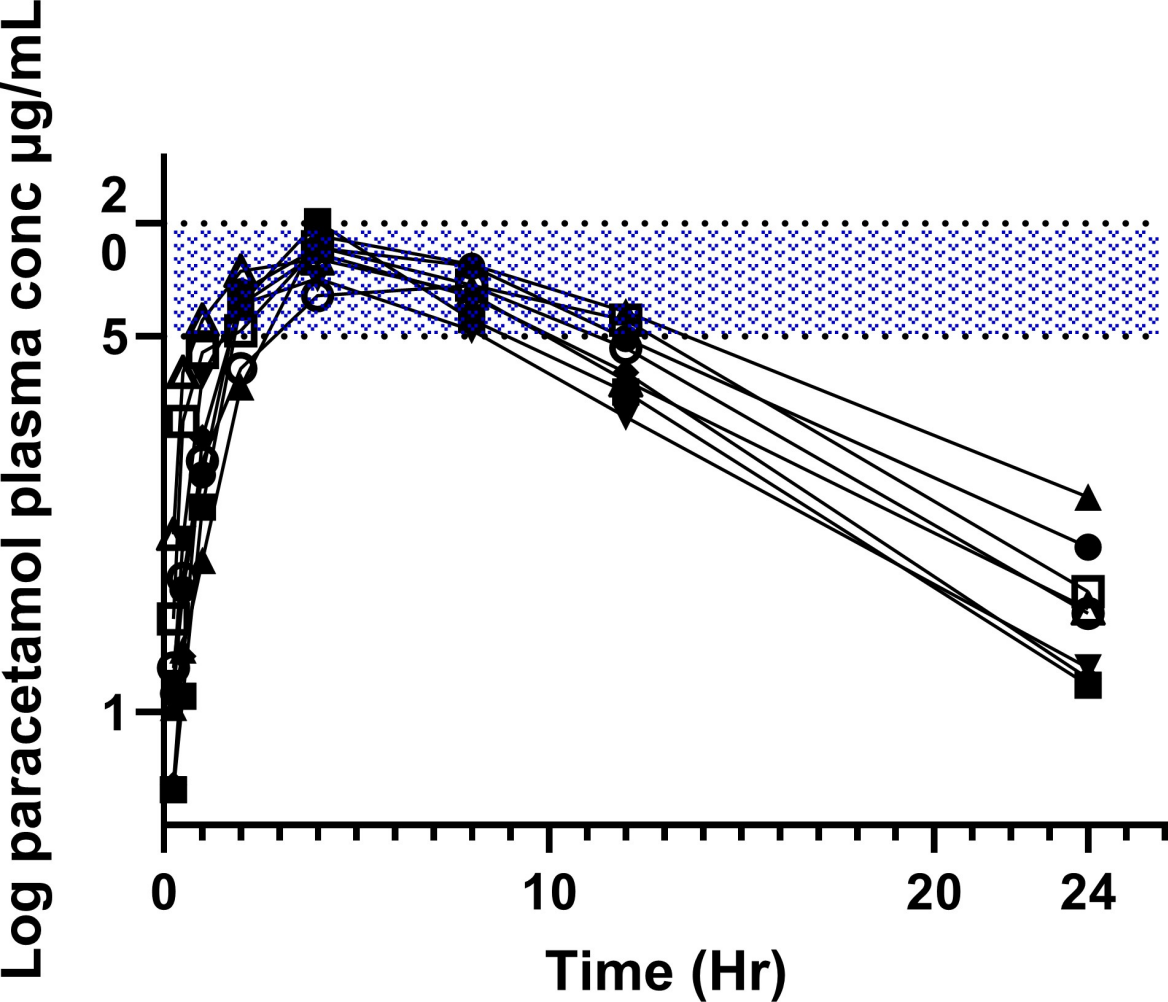
Semi log graph of paracetamol plasma concentration versus time over the first 24 hours, after oral administration of 15mg/kg to eight koalas (repeated in two koalas [K2 and K4]. The hatched zone is the suggested plasma therapeutic concentration range in humans [8, 23, 24].

**Fig 4:**
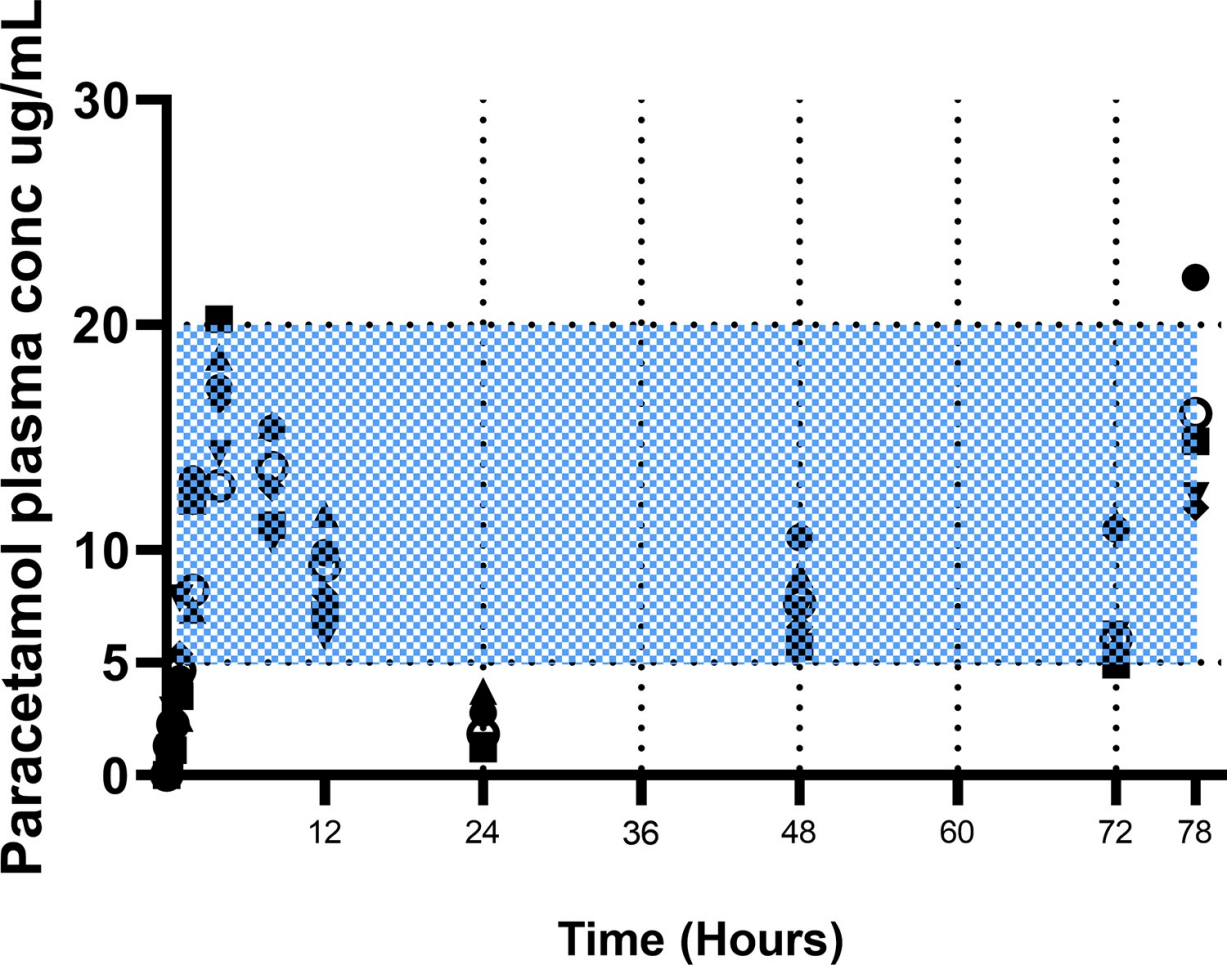
Paracetamol concentration versus time for the oral administration of 15 mg/kg to six koalas for the first 24 hours, followed by trough concentrations just prior to 24, 48 and 72 hour doses. The 78 hour plasma concentrations are also presented. Each shape represents the same koala’s plasma concentrations. The hatched zone is the suggested plasma therapeutic range in humans [8, 23, 24].

S3 Table provides the paracetamol concentrations over time for the 15 mg/kg single s.c. injection (in 2 koalas), for two koalas administered a single oral dose at 15 mg/kg and for the six koalas administered using oral formulation and then after 24 hrs administered every 12 hrs for five additional doses. However, the log paracetamol concentration versus time curves for the oral administration at 15 mg/kg to eight koalas for the first 24 hrs are presented in Fig 3. Fig 4 also shows the paracetamol trough concentrations just prior to the 24, 48 and 72 hour doses.

The pharmacokinetic indices of paracetamol when administered orally and subcutaneously, both at 15 mg/kg over 24 hrs are provided in Table 1.

**Table 1:** Pharmacokinetic indices of paracetamol when administered orally and subcutaneously, both at 15 mg/kg over 24 hrs.

		Single subcutaneous bolus injection of 15 mg/kg			Single oral administration of 15 mg/kg								
Indice	Unit	K1	K2	Median	K3	K4	K5	K6	K7	K8	K2	K4	Median
**ka**	1/hr	5.729	1.588	3.659	0.295	0.691	0.194	0.225	0.159	0.323	0.215	0.175	0.220
**k**_**e**_	1/hr	0.144	0.123	0.134	0.123	0.120	0.107	0.142	0.090	0.129	0.149	0.128	0.125
**t1/2**	hr	4.823	5.616	5.220	5.658	5.788	6.449	4.889	7.669	5.363	4.661	5.423	5.54
**T**_**max**_	hr	0.5	1.0	0.8	4.0	4.0	4.0	4.0	4.0	4.0	4.0	8.0	4.0
**C**_**max**_	μg/mL	15.02	19.61	17.32	17.33	16.10	17.21	20.25	18.59	14.30	16.64	13.66	16.93
**AUC**_**0-24**_	μg/mL*hr	124.53	160.49	142.51	216.86	198.07	223.14	178.11	237.72	158.52	182.32	183.55	190.81
**AUC**_**0-∞**_	μg/mL*hr	128.87	170.15	149.51	233.91	213.79	248.73	186.40	279.05	168.66	190.57	197.90	205.84
**AUC 0-24/0-**_**∞**_	%	0.97	0.90	0.95	0.90	0.90	0.90	0.96	0.90	0.90	0.96	0.90	0.90
**AUMC**_**0-∞**_	μg/mL*h^2^	961.38	1454.97	1207.68	2569.71	2187.58	2964.55	1729.49	3894.82	1646.24	1813.98	2214.62	2201.1
**MRT**_**0-∞**_	h	7.460	8.545	8.003	10.986	10.232	11.919	9.279	14.957	9.761	9.513	11.191	10.609
**Vz/F**	L/kg	0.810	0.714	0.762	0.523	0.586	0.561	0.568	0.595	0.688	0.593	0.586	0.688
**Cl/F**	L/kg /h	0.116	0.088	0.102	0.064	0.070	0.060	0.080	0.054	0.089	0.076	0.070	0.089

Table abbreviations: k_a_ = oral absorption constant; k_e_ = elimination rate constant; t_1/2_ = elimination half-life; T_max_ time to reach maximal plasma concentration; C_max_ = maximal plasma concentration; AUC_0-24 hrs_ = log linear area under the paracetamol plasma concentration time curve form 0 to 24 hrs; AUC_0-**∞**_ = log linear area under the paracetamol plasma concentration time curve form 0 hr to ∞ hrs; AUMC_0-24h_ = area under the moment curve for 24 hrs after paracetamol dosing; MRT = mean residence time; Vz/F apparent volume of distribution, Cl/F = clearance. Koalas K2 and K4 were dosed twice.

The accumulation of paracetamol between the peak concentrations after dosing at 48 and 72 hrs was 1.2.

### Percentage of paracetamol bound to plasma proteins

The mean ± SD percentage of paracetamol at 15 μg/mL (equivalent to the C_max_), 30 μg/mL and 150 μg/mL bound to koala plasma proteins was 61.23 ± 7.46% (4 replicates), 53.20 ±0.18% (n = 2 replicates) and 52.16 ± 11.86% (n = 2 replicates), respectively. The non-specific binding was < 5%.

#### Paracetamol metabolites

**Fig 5:**
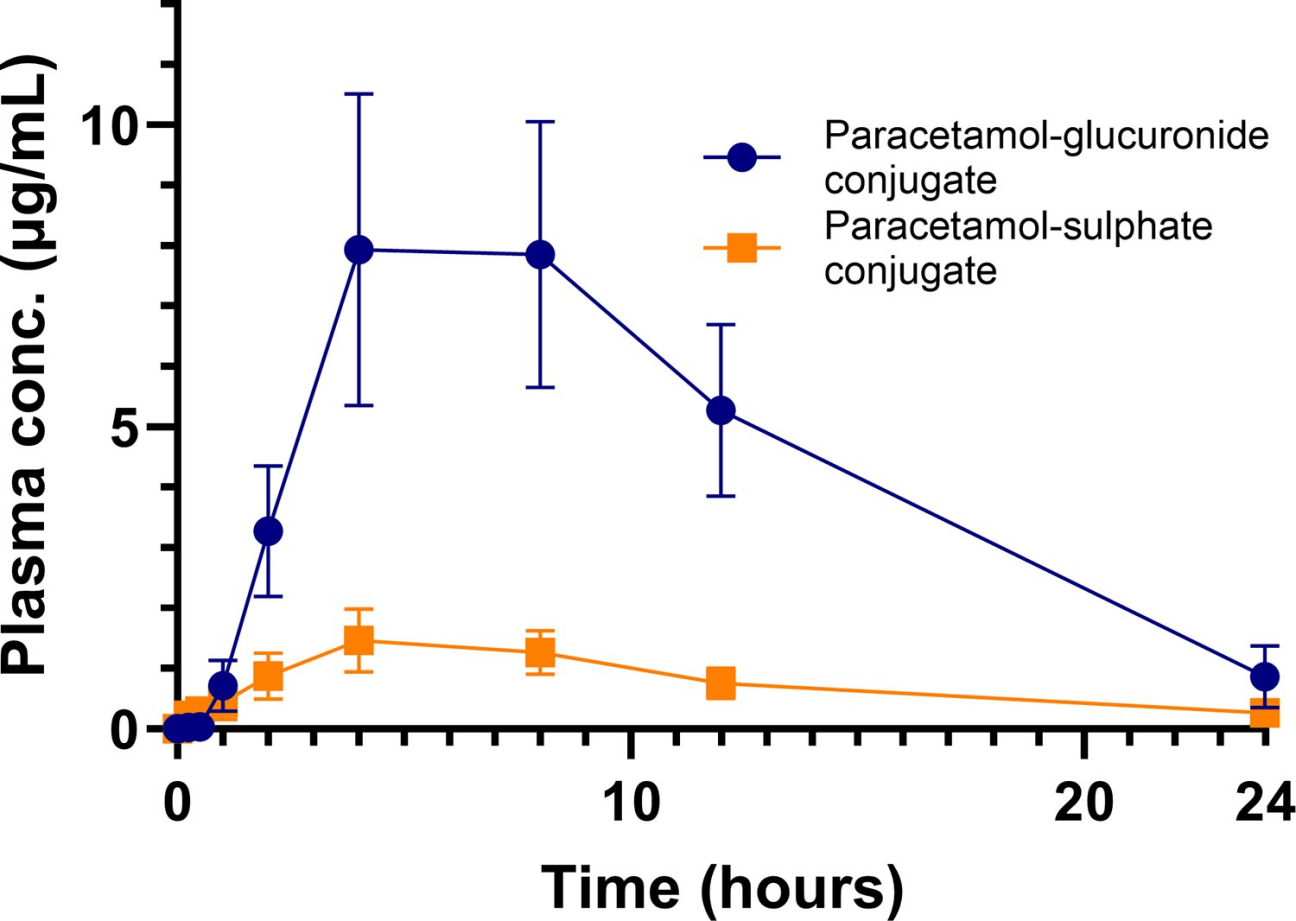
Graphs of paracetamol-glucuronide (PG) and paracetamol-sulphate (PS) plasma concentrations versus time over the first 24 hours after oral administration of 15 mg/kg to six koalas.

The PG and PS plasma concentrations (μg/mL) over time (hrs) for the six koalas administered the oral formulation at 15 mg/kg for 24 hrs, administered every 12 hrs for five additional doses are provided in S4 Table and the change in PG and PS in six koalas after the first dosing, over the first 24 hrs is provided graphically in Fig 5.

### In vitro microsome studies to identify the presence of NAPQI and the rate of paracetamol-glucuronide formation

#### Phase I in vitro study

The substrate depletion of paracetamol at concentrations of 1 μM and 50 μM were only performed with microsomes from the common brush-tailed possum because these enzymes have superior activity to those of other marsupials [4, 25]. Initially paracetamol appeared to undergo rapid depletion for the first 10 minutes and then depleted slowly from minute 10 to minute 30. Two unknown peaks were formed during the incubation however neither could be identified as NAPQI.

#### Phase II in vitro glucuronidation assay

**Fig 6:**
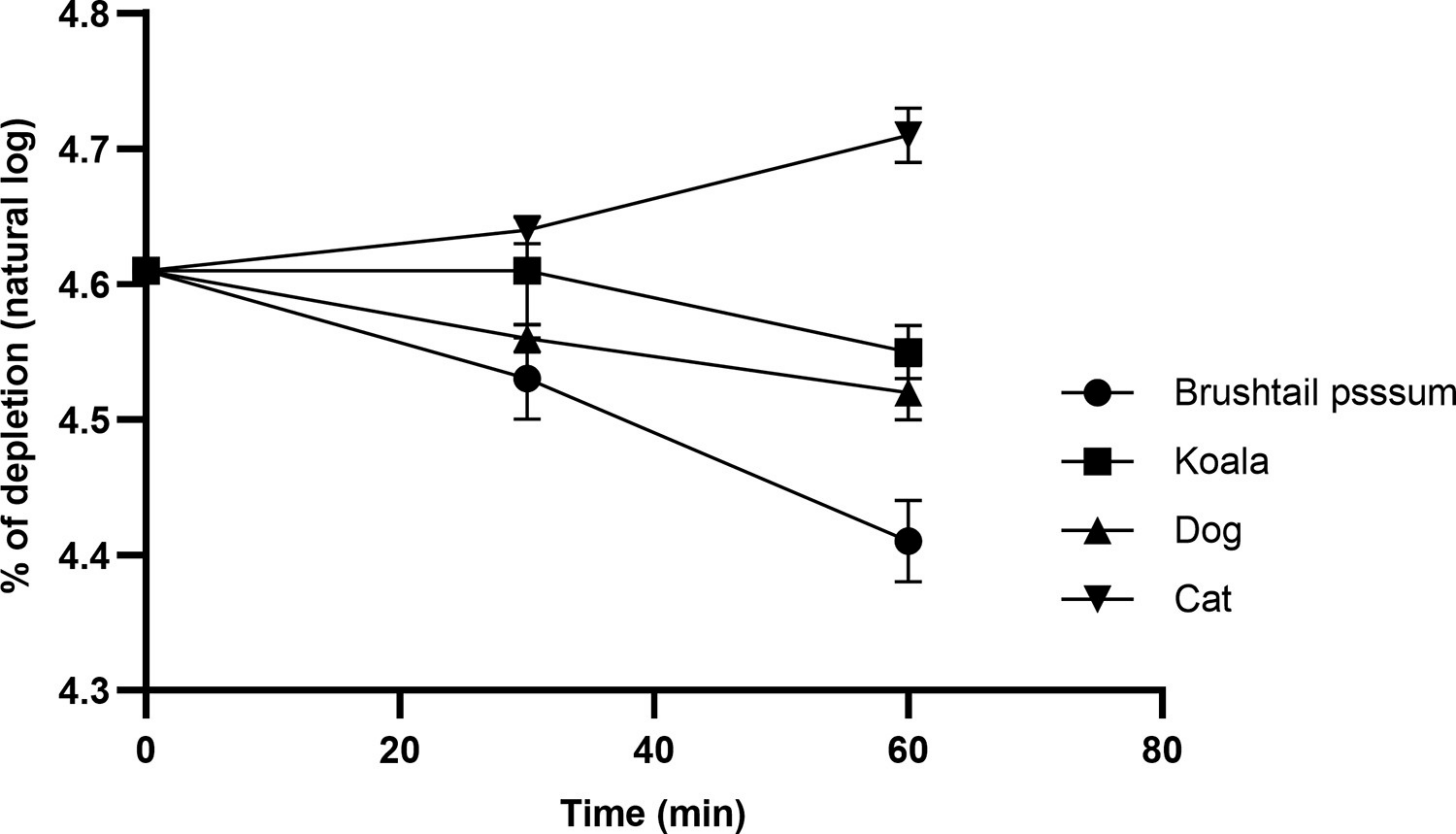
Percentage of depletion of paracetamol as a natural log over 60 minutes, at a concentration of 2.5 μM with 0.5 mg/mL of pooled microsomes from each species.

The percentage of substrate depletion of paracetamol at a concentration of 2.5 μM with 0.5 mg/mL of pooled microsomes from each species is provided in Fig 6 and the numerical data in S5 Table.

**Fig 7:**
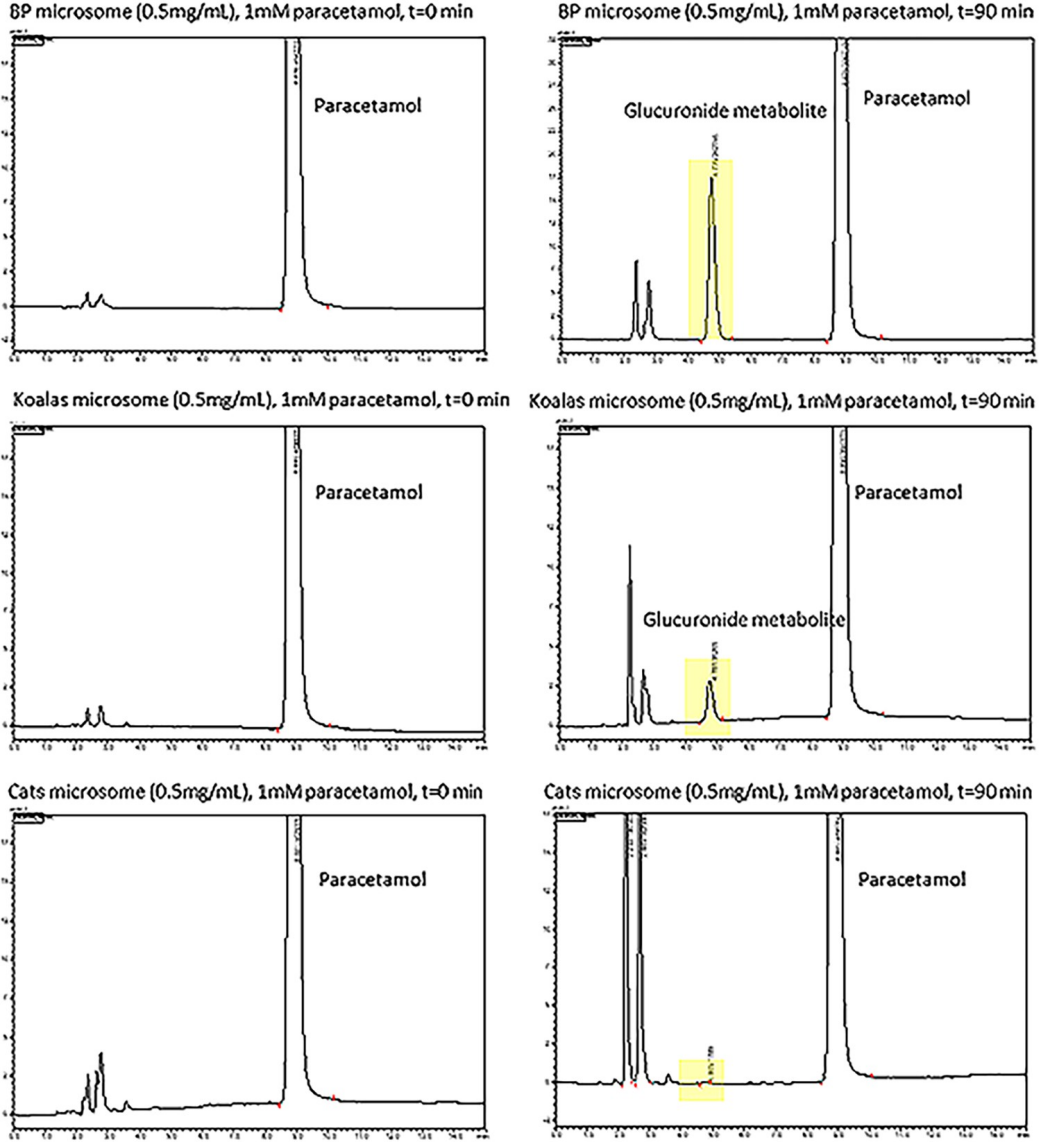
Chromatograms that demonstrate the absence of the glucuronide metabolite when paracetamol is incubated with common brush-tailed possum, koala or feline microsomes at t = 0 minutes. In contrast to the feline microsome incubation, only the common brush-tailed possum and koala microsome incubations show the formation of the glucuronide metabolite at t = 90 minutes. BP = incubated with common brush-tailed possum microsomes.

The *in vitro* half-life and *in vitro* intrinsic clearance (*in vitro* Cl_int_) of paracetamol when incubated with 0.5 mg/mL of microsomes for each species and a phase II glucuronidation assay is provided in Table 2. When incubated with microsomes from the brush-tailed possum, dogs and those of the koala, the substrate depletion was 32%, 10% and 5%, respectively over the 60 min incubation period, the incubation with the feline microsomes resulted in no obvious depletion. Fig 7 demonstrates that when paracetamol is incubated with the microsomes of the common brush-tailed possum and the koala that the glucuronide metabolite is generated, but this metabolite could not be visualised when paracetamol was incubated with feline microsomes.

**Table 2:** Mean ± SD *in vitro* half-life and *in vitro* Cl_int_ of paracetamol undergoing a phase II glucuronidation assay (run in duplicates with each species’ microsomes).

	Units	Microsomes			
		Common brush-tailed possum	Koala	Dog	Cat
***In vitro* t**_**1/2**_	min	220.05 ± 38.90	810.17 ± 254.61	470.46 ± 88.71	No depletion
***In vitro* Cl**_**int**_	μL/min/mg	6.40 ± 1.13	1.80 ± 0.57	3.00 ± 0.57	No depletion

## Discussion

Paracetamol, an over the counter (OTC) medication in Australia, has been administered orally to koalas for decades to provide mild analgesia [12]. However, this is the first study to describe the PK profile in the koala; and using the plasma therapeutic concentration in people, predict the likely efficacy of this dosage. Some orally administered drugs have relatively poor oral absorption in the koala compared to that in cats and dogs (such as meloxicam [3] and enrofloxacin [15]). Therefore, a pilot study medicated two koalas with a 15 mg/kg s.c. injection, and another two with the same dose administered orally, to compare the different administration routes’ ability to undergo absorption. When the median oral AUC_0-∞_ (223.85 μg/mL*hr) was slightly superior to the s.c. AUC_0-∞_ (149.51 μg/mL*hr) it was decided to focus on the oral PK profile as this is the conventional, practical and painless route to administer paracetamol to koalas. There was some initial concern that any injection route could result in soft tissue injury [14]; therefore the intravenous formulation was diluted 50:50 with isotonic saline as the s.c. injection and there were no observed adverse effects on the animals.

Paracetamol is the most widely used analgesic in people, globally [8], however paracetamol is not a popular veterinary medicine, probably a result of its administration to cats, which even at low doses results in a toxic metabolite [26], and there are many efficacious NSAIDs available for companion animals, especially dogs. The toxic doses for cats, dogs, humans and rats are provided in Table 3. Paracetamol has some mechanistic similarities to NSAIDs, whereby it inhibits COX-2 in the central nervous system, However, in contrast to NSAIDs, paracetamol is considered to have no anti-inflammatory activity [27].

**Table 3:** Paracetamol oral toxic dose for cat, dog, human and rat.

`	Toxic dose
**Cat**	There is no safe dose of paracetamol for cats. The toxic dose is reported as 50 to 100 mg/kg [26]
**Dog**	Clinical signs of toxicity seen with doses in excess of 200 mg/kg body-weight (BW) [28]
**Human**	Toxicity develops from > 170 mg/kg (12 gm per adult); 200 mg/kg for children [8, 29, 30]
**Rat**	Lethal dose (LD) _50(100 days)_ ± S.E. (oral dose of paracetamol that killed 50% of young male albino rats when given daily for 100 days) = 0.77 ± 0.02 g/kg per day [31]

### Paracetamol PK profile

There are PK profiles of paracetamol described for some animals such as dogs [32–36] and horses [37, 38]. It is problematic to compare apparent clearance between species when there is no intravenous dosing PK information for the koala. However, the oral half-life in the koala (median [range] is longer than in the dog (1.25 hrs [when fed]) [35], the horse (mean ± SD 2.95 ± 0.62 hrs Day 7; 4.64 ± 3.56 hrs Day 21) [37, 38], and in humans (1.9–2.5 hrs) [39]. The percentage of drug bound to plasma proteins is reported as 49.2 ± 6.80 [38] or 52.3 ± 8.6% [37] in the horse and between 15% and 21% for both pigs and humans, respectively [40], and 11.0 ± 1.2% in the dog [41]. In contrast, a concentration of 15 μg/mL (similar to plasma C_max_) has a percentage of bound drug of 61.23 ± 7.46% in the koala and this may account for the longer half-life in this species. Earlier studies reported that paracetamol does not bind to plasma proteins in humans [23, 42] however 24.1% of bound drug has been reported in humans with a trend toward less binding at higher drug concentrations [43]. The unbound form is the active drug component and the human: koala unbound drug fraction (fu) ratio is approximately 80: 40, which infers that the total human plasma concentration has 2-fold more “active drug” than an equivalent total plasma concentration in the koala. When the concentration was 150 μg/mL the binding was reduced to 52.16 ± 11.86% in koala plasma, similar to what has been observed in humans, i.e. an increase in drug concentration decreases the bound drug percentage [43].

### Paracetamol dosage predicted efficacy

To estimate whether the oral paracetamol dosage resulted in plasma paracetamol concentrations that could achieve therapeutic analgesic efficacy, plasma concentration in the koala was compared to the plasma therapeutic range in humans, suggested between 4–20 μg/mL [8, 23, 24, 44]. When paracetamol was administered orally at 15 mg/kg over the first 24 hrs the plasma concentration remained in the human therapeutic concentration range for approximately 10 hrs (see S3 Table), with a median (range) C_max_ of 16.93 μg/mL (13.66–20.25 μg/mL). When administered at the same dose twice daily, the trough concentrations directly prior to the 48 hrs and 72 hrs doses had a median (range) plasma concentration of 7.50 μg/mL (5.70–10.55 μg/mL) and 6.16 μg/mL (4.90–10.94 μg/mL) respectively, illustrated in Fig 4. However, as explained above, the total plasma concentrations may be comparable, but the unbound/active drug concentrations are not.

At a twice daily oral dose at 15 mg/kg, the paracetamol accumulation factor was 1.2. A factor of 1 or less indicates no accumulation and 1.2 is considered within the ‘no-accumulation” limit [45].

Based on a median half-life of 5.5 hrs, steady state should be reached in five half-lives at around 27.5 hrs of dosing. The oral bioavailability of paracetamol in humans ranges from 0.63 to 0.89 [46], however, this study was unable to establish the oral bioavailability in the koala.

Prior to 1^st^ February 2018, paracetamol and codeine formulations were available OTC in Australia. Now all codeine formulations are prescription only. Paracetamol-codeine combinations have been shown to have a small but statistically significant difference, compared to paracetamol alone with respect to the analgesic effects in people particularly on a single administration [47], however with repeated administration the combination increases the occurrence of side effects in people such as dizziness, drowsiness, nausea, vomiting and constipation [47].

### Paracetamol metabolism

In the koala there is some evidence that some NSAIDs such as meloxicam undergo phase I metabolism by cytochrome P450 CYP2C-like enzymes and are rapidly eliminated [4]. NSAID oxidation by CYP2C enzymes is very efficient in the koala [48]. It is hypothesized that this is a key pathway for metabolizing terpenoids that make up around 16% of their almost exclusive eucalypt diet [49]. In contrast, paracetamol undergoes phase I metabolism by CYP2E so the median (range) oral half-life of 5.5 hrs (4.7–7.7 hrs) exceeds that of the IV half-life of the NSAID meloxicam of 1.19 h (range 0.71–1.62 h) in the koala [3].

Paracetamol undergoes phase II metabolism by both glucuronidation and sulfation in many species [8, 33, 35, 37]. The reason for paracetamol toxicity in the cat is that the toxic phase I metabolite (NAPQI) cannot undergo phase II conjugation due to the lack of UDP-glucuronosyltransferase (UGT) enzymes [9]. This investigation attempted a phase I *in vitro* study to ascertain the amount of NAPQI generated when paracetamol is incubated with common-brush-tailed possum microsomes (very active microsomes [4]) and an NADPH energy system and cofactors, but the analytical NAPQI standard rapidly transformed into paracetamol, indicating instability and thus this investigation was discontinued. However, the *in vitro* phase II reaction was more successful with the paracetamol being converted to the glucuronide metabolite within 90 minutes of incubation with either common brush-tailed possum or koala microsomes. Running a phase II reaction simultaneously with feline microsomes, not surprisingly the glucuronide metabolite was not generated when paracetamol was incubated with feline microsomes. The rate of glucuronide formation and the formation of the glucuronide when paracetamol was incubated with microsomes of various species are illustrated in Figs 5 and 6, respectively. The *in vitro* depletion of paracetamol to the glucuronide metabolite is further supported by the first detection of the glucuronide metabolite as well as the sulphate metabolite in the plasma of all koalas, 30 minutes and 15 minutes, respectively, after oral dosing, and then detectable at all subsequent time points over the first 24 hrs as illustrated in Fig 4.

### Repeated dosing effect on biochemical analytes

A review of the long-term effects of paracetamol treatment, at therapeutic dosage, in people reports no evidence for hepatoxicity, either in healthy individuals or those with chronic liver disease, with the exception of those in a poor nutritional state [7]. This same review also concluded that there was insufficient evidence that paracetamol administration was associated with nephropathy. Our koala study compared the changes in plasma biochemical analyte and electrolyte values prior to, and after five, doses. All electrolytes and biochemical analytes were within their normal ranges on both occasions. The only significant differences were in ALT and total plasma protein (p < 0.03 for both). ALT is an enzyme primarily found in the liver. Plasma or serum elevations of ALT may be seen with drugs that cause hepatotoxicity and other liver diseases [50]. A post-treatment rise in ALT values (median change +4 U/L) was observed in all six koalas. Repeated therapeutic paracetamol administration to human adults is also known to result in a transient elevation of ALT but in healthy adults there is no progression to hepatotoxicity [7]. Three koalas had a decrease in total protein (range of difference—1 to– 9 g/L), there was a decrease in globulin values in four of the koalas (range -1 to -3 g/L) but this was not significant. A similar protein pattern has been reported in rats administered a dose of paracetamol (171.41 mg/kg a day for 14 days), when compared to the control group, and there was also a significant decrease in total plasma proteins and globulins (p <0.05 for both), with the albumin concentrations remaining constant [51].

The formulation of oral paracetamol administered to the koalas contains the excipients hydroxybenzoates, saccharin, benzoic acid, potassium sorbate and sorbitol, the authors cannot confirm whether it was repeat dosing of one or more of these excipients that may have elevated the ALT and/or decreased the total plasma protein concentration.

This study has limitations including that there are limited numbers of Taronga zoo’s captive collection that are available at any one time for recruitment to these studies. Likewise it is not possible to correlate the paracetamol plasma concentrations with likely analgesic activity in this species as only clinically normal animals were recruited. Therefore the koala’s paracetamol plasma concentrations were compared to the therapeutic analgesic range reported for humans as the therapeutic plasma concentrations that achieve an “effect” are more likely to have greater similarity between species, than the PK profile [52]. Also, determination of the percentage of the drug bound to the plasma proteins *in vitro* may deviate from that in the live animal as *in vivo* protein binding may also be affected by not only protein concentration, but also the structure of the plasma proteins and their affinity to bind substrates which can vary with age, disease and /or presence of competing endogenous and exogenous compounds such as dietary constituents or other therapeutic drugs [53, 54]. The plasma used in this study was also initially frozen, then thawed and adjusted to physiological pH, this can also affect the % bound compared to when “fresh” plasma is used [55]. The proportion of drug binding to plasma protein is also influenced by the drug concentration, the plasma protein concentration, competition with other drugs for protein binding sites and the attraction for, or strength of bonding, i.e. the paracetamol’s affinity for the drug and the plasma proteins. This study is not able to comment on the paracetamol affinity for koala plasma proteins. Paracetamol has been shown to be primarily bound to albumin in humans [43].

## Conclusion

Paracetamol at an oral dose of 15 mg/kg administered twice daily is absorbed in the koala and reaches the same total therapeutic plasma concentration range that is suggested to provide a mild to moderate analgesic effect in humans. However, the difference in the plasma protein binding means that there is significantly less active proportion of drug in the koala. When administered at this dose twice daily for five doses all biochemical analytes and electrolytes remained within reference ranges however at the end of dosing a slight but statistical elevation in ALT was evident as was a decrease in total protein. A slight elevation in the dose may be warranted to increase the more active drug available as in humans the proportion of free drug increases with increasing dose [43], however such a dose for the koala requires further modelling and clinical monitoring. A preferable solution may involve administering the current dosage in combination with another synergistic analgesic such as tramadol as a subcutaneous injection [1].

## Supporting information

S1 Table Validation of the QC paracetamol, paracetamol-glucuronide and paracetamol-sulphate samples in koala plasma for accuracy and precision on three occasions. (DOCX)

S2 Table Weight, age and sex of the koalas (K). (DOCX)

S3 Table Paracetamol plasma concentrations (μg/mL) over time (hrs) for the 15 mg/kg single subcutaneous injection (in two koalas), and for two koalas administered a single oral dose at 15 mg/kg, and for the six koalas administered the oral formulation at 15 mg/kg; and after 24 hrs—administered every 12 hrs for five additional doses. Grey cells over the first 24 hrs signify when the plasma concentration is within the human therapeutic range of 4–20 μm/mL.
(DOCX)

S4 Table Paracetamol-glucuronide and paracetamol-sulphate plasma concentrations (μg/mL) over time (hrs) for the six koalas administered the oral formulation at 15 mg/kg and after 24 hrs, administered every 12 hrs for five additional doses. (DOCX)

S5 Table Mean ± SD percentage (as natural logs) of paracetamol depletion when incubated with pooled common brush-tailed possum, koala, dog and cat microsomes at 0, 30 and 60 minutes as represented in Fig 6. Each time-point was run as duplicates.
(DOCX)
