# Supplementary material for: Pharmacokinetic profile of oral and subcutaneous administration of paracetamol in the koala (Phascolarctos cinereus) and prediction of its analgesic efficacy
Source: PLoS One. 2024 Apr 17;19(4):e0300703. doi: 10.1371/journal.pone.0300703 (PMC11023281; doi:10.1371/journal.pone.0300703)
Supplement: S1 Table — (DOCX) [file pone.0300703.s001.docx]

**S1 Table. Validation of the QC paracetamol, paracetamol-glucuronide and paracetamol-sulphate samples in koala plasma for accuracy and precision on three occasions.**

|  | **Paracetamol** | | **Paracetamol-glucuronide** | | **Paracetamol-sulphate** | |
| --- | --- | --- | --- | --- | --- | --- |
| **Nominal conc.* µg/mL** | Mean observed conc. +/- SD µg/mL  (n = 3) | Precision  (CV %)  Accuracy  (Range%)  (n = 3) | Mean observed conc. +/- SD µg/mL (n = 3) | Precision  (CV %)  Accuracy  (Range%)  (n = 3) | Mean observed conc. +/- SD µg/mL  (n = 3) | Precision  (CV %)  Accuracy  (Range%)  (n = 3) |
| **20** | 19.96 ± 0.61 | 3.08  (96.25-101.69) | 19.89 ± 0.72 | 3.60  (95.31-101.73) | Not done |  |
| **10** | 10.03 ± 0.52 | 5.23  (95.95-106.10) | 10.24 ± 0.38 | 3.71  (98.23-105.72) | 10.7 ± 0.33 | 3.04  (103.30-109.44) |
| **5** | 5.07 ± 0.12 | 2.43  (99.56-104.16) | 4.98 ± 0.08 | 1.53  (98.34-101.22) | 5.14 ± 0.35 | 6.80  (88.44-103.41) |
| **1.25** | 1.24 ± 0.01 | 0.73  (98.79-100.19) | 1.23 ± 0.06 | 4.46  (93.54-101.71) | 1.25 ± 0.08 | 6.06  (95.19-106.76) |
| **0.625** | 0.56 ± 0.00 | 0.34  (89.50-90.08) | 0.58 ± 0.43 | 7.42  (85.54-99.20) | 0.59 ± 0.08 | 12.90  (85.78-107.88) |

Precision (expressed as the coefficient of variation [CV] %) was calculated by dividing the standard deviation (SD) by the mean and multiplied by 100. Accuracy was calculated by observed concentration divided by nominal concentration and multiple by 100.

*concentration
