# Supplementary material for: Pharmacokinetic profile of oral and subcutaneous administration of paracetamol in the koala (Phascolarctos cinereus) and prediction of its analgesic efficacy
Source: PLoS One. 2024 Apr 17;19(4):e0300703. doi: 10.1371/journal.pone.0300703 (PMC11023281; doi:10.1371/journal.pone.0300703)
Supplement: S2 Table — (DOCX) [file pone.0300703.s002.docx]

**S2 Table. Weight, age and sex of the koalas (K).**

|  | **K1** | **K2** | **K3** | **K4** | **K5** | **K6** | **K7** | **K8** |
| --- | --- | --- | --- | --- | --- | --- | --- | --- |
| **Weight (kg)** | 7.6 | 10.3 | 8 | 8.7 | 7.2 | 7.4 | 7.8 | 9.6 |
| **Age (years)** | 5.4 | 5 | 3.8 | 4.83 | 5.83 | 2.8 | 3.8 | 7.5 |
| **Sex** | Female | Male | Male | Male | Female | Female | Female | Male |

K2 and K4 were medicated for this study ten and seven months apart, respectively.
