# Supplementary material for: Pharmacokinetic profile of oral and subcutaneous administration of paracetamol in the koala (Phascolarctos cinereus) and prediction of its analgesic efficacy
Source: PLoS One. 2024 Apr 17;19(4):e0300703. doi: 10.1371/journal.pone.0300703 (PMC11023281; doi:10.1371/journal.pone.0300703)
Supplement: S3 Table — Grey cells over the first 24 hrs signify when the plasma concentration is within the human therapeutic range of 4–20 μm/mL. (DOCX) [file pone.0300703.s003.docx]

**S3 Table. Paracetamol plasma concentrations (µg/mL) over time (hrs) for the 15 mg/kg single subcutaneous injection (in two koalas), and for two koalas administered a single oral dose at 15 mg/kg, and for the six koalas administered the oral formulation at 15 mg/kg; and after 24 hrs - administered every 12 hrs for five additional doses. Grey cells over the first 24 hrs signify when the plasma concentration is within the human therapeutic range of 4 – 20 µm/mL.**

|  | **Single 15 mg/kg subcutaneous bolus injection** | | **Single oral administration 15 mg/kg** | | **Initial single oral administration 15 mg/kg; after 24 hrs 15 mg/kg was administered orally every 12 hrs for five additional doses** | | | | | |
| --- | --- | --- | --- | --- | --- | --- | --- | --- | --- | --- |
| **Time (hrs)** | K1 | K2 | K3 | K4 | K5 | K6 | K7 | K8 | K2 | K4 |
| **0** | 0 | 0 | 0 | 0 | 0 | 0 | 0 | 0 | 0 | 0 |
| **0.25** | 12.59 | 7.06 | 1.77 | 2.97 | 1.12 | 0.62 | 1.03 | 1.19 | 0.64 | 1.31 |
| **0.5** | 15.02 | 12.47 | 9.06 | 8.03 | 2.12 | 1.10 | 1.46 | 2.88 | 1.41 | 2.27 |
| **1** | 14.98 | 19.61 | 5.94 | 11.06 | 4.28 | 3.52 | 2.53 | 7.87 | 5.39 | 4.66 |
| **2** | 13.06 | 19.09 | 10.38 | 14.93 | 13.16 | 12.19 | 7.34 | 12.15 | 12.16 | 8.22 |
| **4** | 11.37 | 13.75 | 17.33 | 16.10 | 17.21 | 20.25 | 18.59 | 14.29 | 16.64 | 12.86 |
| **8** | 5.86 | 7.23 | 13.72 | 12.99 | 15.42 | 11.11 | 15.55 | 10.41 | 12.76 | 13.66 |
| **12** | 3.84 | 5.10 | 11.05 | 7.63 | 9.88 | 7.12 | 11.62 | 6.11 | 8.02 | 9.32 |
| **24** | 0.62 | 1.19 | 2.09 | 1.88 | 2.75 | 1.18 | 3.74 | 1.31 | 1.23 | 1.83 |
| **Dose 2 after 24 hrs** | | | | | | | | | | |
| **Dose 3 at 36 hrs** | | | | | | | | | | |
| **48** |  | | | | 10.55 | 5.70 | 8.93 | 7.35 | 6.59 | 7.64 |
| **Dose 4 after 48 hrs** | | | | | | | | | | |
| **Dose 5 at 60 hrs** | | | | | | | | | | |
| **72** |  | | | | 10.94 | 4.90 | 11.24 | 6.27 | 5.59 | 6.06 |
| **Dose 6 after 72 hrs** | | | | | | | | | | |
| **78** |  | | | | 22.11 | 14.84 | 31.64 | 12.41 | 11.88 | 16.05 |
