# Supplementary material for: Pharmacokinetic profile of oral and subcutaneous administration of paracetamol in the koala (Phascolarctos cinereus) and prediction of its analgesic efficacy
Source: PLoS One. 2024 Apr 17;19(4):e0300703. doi: 10.1371/journal.pone.0300703 (PMC11023281; doi:10.1371/journal.pone.0300703)
Supplement: S4 Table — (DOCX) [file pone.0300703.s004.docx]

**S4 Table: Paracetamol-glucuronide and paracetamol-sulphate plasma concentrations (µg/mL) over time (hrs) for the six koalas administered the oral formulation at 15 mg/kg and after 24 hrs, administered every 12 hrs for five additional doses.**

|  | **Paracetamol-glucuronide plasma concentration (µg/mL)** | | | | | | **Paracetamol-sulphate plasma concentration (µg/mL)** | | | | | |
| --- | --- | --- | --- | --- | --- | --- | --- | --- | --- | --- | --- | --- |
| **Time (hrs)** | K5 | K6 | K7 | K8 | K2 | K4 | K5 | K6 | K7 | K8 | K2 | K4 |
| **0.25** | 0.00 | 0.00 | 0.00 | 0.00 | 0.00 | 0.14 | 0.00 | 0.00 | 0.00 | 0.00 | 0.00 | 0.00 |
| **0.5** | 0.00 | 0.00 | 0.00 | 0.00 | 0.00 | 0.22 | 0.25 | 0.68 | 0.13 | 0.37 | 0.87 | 0.32 |
| **1** | 0.50 | 0.57 | 0.06 | 1.22 | 1.09 | 0.87 | 0.69 | 1.32 | 0.45 | 0.77 | 1.39 | 0.62 |
| **2** | 3.69 | 4.45 | 1.76 | 3.28 | 4.22 | 2.23 | 1.57 | 2.24 | 1.38 | 0.88 | 1.79 | 0.94 |
| **4** | 9.65 | 11.94 | 6.72 | 7.39 | 7.48 | 4.43 | 1.46 | 1.63 | 1.31 | 0.64 | 1.49 | 1.07 |
| **8** | 11.36 | 8.56 | 8.65 | 5.52 | 7.45 | 5.56 | 0.82 | 1.18 | 0.77 | 0.41 | 0.74 | 0.63 |
| **12** | 7.71 | 5.53 | 5.72 | 3.66 | 4.82 | 4.23 | 0.19 | 0.51 | 0.14 | 0.24 | 0.25 | 0.24 |
| **24** | 1.66 | 0.36 | 1.31 | 0.51 | 0.63 | 0.74 | 0.00 | 0.00 | 0.00 | 0.00 | 0.00 | 0.00 |
| **Dose 2 after 24 hrs** | | | | | | | | | | | | |
| **Dose 3 at 36 hrs** | | | | | | | | | | | | |
| **48** | 8.25 | 3.93 | 4.36 | 3.73 | 4.23 | 3.05 | 0.99 | 0.76 | 0.85 | 0.76 | 0.44 | 0.37 |
| Dose 4 after 48 hrs | | | | | | | | | | | | |
| Dose 5 at 60 hrs | | | | | | | | | | | | |
| **72** | 9.94 | 3.41 | 5.13 | 3.31 | 3.74 | 3.15 | 1.69 | 0.65 | 0.65 | 0.92 | 0.98 | 0.29 |
| **Dose 6 after 72 hrs** | | | | | | | | | | | | |
| **78** | 17.64 | 8.26 | 13.22 | 8.58 | 8.01 | 7.22 | 2.47 | 0.91 | 1.89 | 0.84 | 2.03 | 0.75 |
