# Supplementary material for: Pharmacokinetic profile of oral and subcutaneous administration of paracetamol in the koala (Phascolarctos cinereus) and prediction of its analgesic efficacy
Source: PLoS One. 2024 Apr 17;19(4):e0300703. doi: 10.1371/journal.pone.0300703 (PMC11023281; doi:10.1371/journal.pone.0300703)
Supplement: S5 Table — Each time-point was run as duplicates. (DOCX) [file pone.0300703.s005.docx]

**S5 Table. Mean ± SD percentage (as natural logs) of paracetamol depletion when incubated with pooled common brush-tailed possum, koala, dog and cat microsomes at 0, 30 and 60 minutes as represented in Fig 6. Each time-point was run as duplicates.**

| **Time (minutes)** | **Mean % of paracetamol depletion as natural log** | **SD % of paracetamol depletion as natural log** |
| --- | --- | --- |
| **Common brush-tailed possum microsomes** |  |  |
| **0** | 4.61 | 0.00 |
| **30** | 4.53 | 0.03 |
| **60** | 4.41 | 0.03 |
| **Koala microsomes** |  |  |
| **0** | 4.61 | 0.00 |
| **30** | 4.61 | 0.04 |
| **60** | 4.55 | 0.02 |
| **Dog microsomes** |  |  |
| **0** | 4.61 | 0.00 |
| **30** | 4.56 | 0.01 |
| **60** | 4.52 | 0.02 |
| **Cat microsomes** |  |  |
| **0** | 4.61 | 0.00 |
| **30** | 4.64 | 0.01 |
| **60** | 4.71 | 0.02 |
